# The role of microbes in the formation of modern and ancient phosphatic mineral deposits

**DOI:** 10.3389/fmicb.2012.00241

**Published:** 2012-07-05

**Authors:** Chris H. Crosby, Jake V. Bailey

**Affiliations:** Department of Earth Sciences, University of Minnesota-Twin Cities,Minneapolis, MN, USA

**Keywords:** apatite, *Beggiatoa*, Doushantuo, phosphorites, polyphosphate, *Thiomargarita*, upwelling

## Abstract

The formation of marine phosphatic mineral deposits remains incompletely understood, despite decades of research. The involvement of bacteria in this process has long been suspected, and both modern and ancient associations between bacteria and phosphorites have been recorded. Only recently has a specific bacterial metabolic process associated with the formation of phosphorites been discovered. Recent studies demonstrate that polyphosphate utilization by sulfide-oxidizing bacteria results in the rapid precipitation of apatite – providing at least a partial mechanism to explain the close spatial correlation between accumulations of sulfide-oxidizing bacteria and modern phosphorites. Possible fossilized bacteria are known from ancient phosphatic mineral deposits. Potentially, the fossilized cells represent the remains of bacteria that induced the formation of those phosphorites. However, robust criteria for the recognition of these bacteria have yet to be identified.

## THE PHOSPHOROUS CYCLE AND PHOSPHATIC MINERAL DEPOSITS

Phosphorus (P) is one of the few elements that all life requires – a constituent of the molecules of genetic information, energy currency, and many membranes of living organisms (Sterner and Elser, 2002). As a limiting nutrient for primary productivity, phosphorus is rapidly recycled in the environment, limiting its accumulation in sediments (e.g., Benitez-Nelson, 2000). While most organic matter and its associated phosphorus is transformed into an inorganic state and recycled in the water column, some organic phosphorus reaches the sediments as particulate organic matter such as phytodetritus and fish debris (Suess, 1981). Additionally, phosphate commonly adsorbs to iron oxyhydroxides, including on the surfaces of clay particles and colloids that are eventually deposited in the sediment (Krom and Berner, 1980; Crosby et al., 1984). As they become buried in sediment, iron minerals encounter a zone in which bacterial dissimilatory iron reduction commonly results in the dissolution of iron oxides and concomitant release of HPO_4_^2-^ (Ruttenberg and Berner, 1993). The ubiquitous degradation of organic compounds and dissolution of Fe-oxides in the sediments thus limit the sedimentary accumulation of P. However, under still not fully constrained conditions, the complex interaction of biological concentration, mineral precipitation, and sedimentary reworking can result in the concentration of phosphorus as phosphorite rock deposits (Cook, 1976; Sheldon, 1981; Glenn et al., 1994; Krajewski et al., 1994; Föllmi, 1996; Schulz and Schulz, 2005; Dornbos, 2010; Filippelli, 2011).

Phosphorites are relatively rare marine sedimentary units containing significant amounts of P resulting from the concentration of the mineral apatite Ca_5_(PO_4_)_3_(F,Cl,OH), or its more complex form carbonate fluorapatite (CFA) (Nathan, 1984). Phosphorites contain 6–18% P_2_O_5_, distinguishing them from most sedimentary rock and marine sediments which generally have less than 0.3 wt% P_2_O_5_ (Van Cappellen and Berner, 1988; Jarvis et al., 1994). Phosphate rock, in a range of morphologies including phosphorite mud, laminae, crusts, pellets, nodules, skeletal fragments, and cements has been recognized in geologic rock strata since the late 1700s (Glenn et al., 1994; Föllmi, 1996; Rakovan, 2002). The discovery of a way to extract phosphate from phosphorites led to heightened interest in the use of phosphate as a fertilizer, where it now accounts for approximately 85% of the world’s phosphate consumption. Phosphorites have now been identified in ancient sedimentary rock strata on every continent, but it was not until the British “Challenger” expedition of 1873–1876 that geologically recent phosphorites were recovered from modern marine sediments of the Agulhas Bank area off South Africa (Murray and Renard, 1891). Since then, extensive phosphorites have been found to be forming under the low-latitude upwelling zones on the western shelves and continental margins of North and South America, Africa, and India, as well as off the east coast of Australia, extending the age of known phosphorite formations from the Proterozoic to the present (Baturin et al., 1972; Parker and Andseisser, 1972; Veeh et al., 1973; Burnett et al., 1980; Sheldon, 1981). But what processes and mechanism(s) are responsible for the formation of these enigmatic geological deposits?

## THE FORMATION OF PHOSPHORITES IN MODERN SEDIMENTS

**FIGURE 1:**
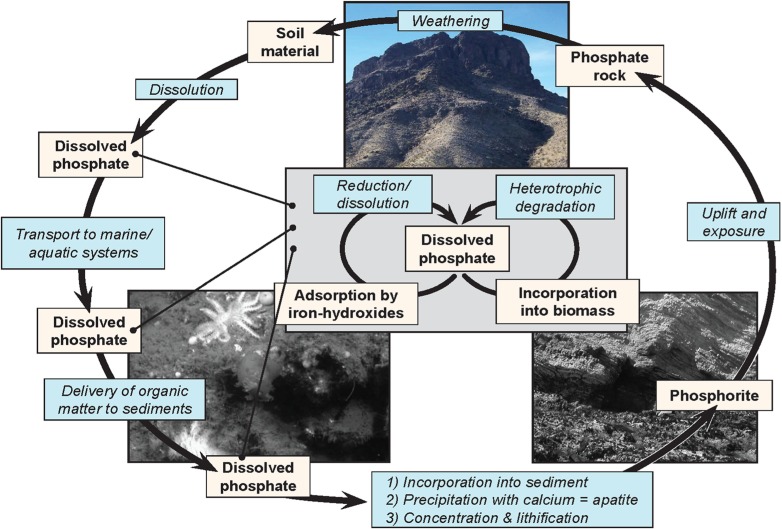
**The phosphorous cycle, showing the complexity of phosphorus cycling, and the relative inaccessibility of phosphorus once it has been lithified**. The inset reflects a cycle through which phosphorus can cycle repeatedly wherever the biosphere can access it.

 Understanding the processes that lead to the deposition of phosphatic minerals is necessary for understanding the distribution and occurrence of this important non-renewable resource and its place within the broader phosphorous cycle (**Figure 1**). The process of phosphogenesis begins with the precipitation of CFA or its metastable precursors within the top few centimeters of marine sediment. It is generally thought that disseminated CFA is then concentrated through a variety of sedimentological processes such as reworking (Cook, 1976; Glenn et al., 1994; Föllmi, 1996; Filippelli, 2011) Concentrated CFA can undergo additional diagenetic transformations such as the precipitation of additional authigenic apatite or carbonate precipitation, perhaps accompanied by multiple cycles of sedimentary reworking (e.g., Baturin cycles and variations thereof). The details of this process are beyond the scope of this review, but see Filippelli (2011) for a recent review.

The majority of modern and ancient phosphorites are associated with sediments beneath coastal upwelling zones and these are the subject of active research and the following discussion. Briefly, in upwelling zones, nutrient-rich waters stimulate primary productivity in the photic zone, resulting in a downward flux of phytodetritus, much of which can accumulate in the sediments due to the high organic flux, high biological oxygen demand, and short sinking transit time on the shallow continental shelf. Some combination of biochemical and geochemical mechanisms then act to release P and inhibit its recycling back into the water column. Pore water conditions that are supersaturated and kinetically favorable for the precipitation of apatite then result in mineral precipitation that proceeds through the initial formation of amorphous apatite precursors or other metastable precursors that eventually transform into CFA (Nancollas et al., 1979; Gunnars et al., 2004; Omelon and Grynpas, 2008).

Various mechanisms have been proposed to produce conditions favoring the precipitation of apatite precursors. One model invoking direct microbial involvement, with both experimental and *in situ* evidence to support it, involves polyphosphate utilization by sulfide-oxidizing bacteria (Schulz and Schulz, 2005). In shelf sediments beneath eutrophic marine waters, such as subtropical eastern boundary current regions, high rates of aerobic remineralization in the water column followed by bacterial dissimilatory sulfate reduction in the sediments lead to oxygen depletion and production of copious hydrogen sulfide in the sediments (Jørgensen, 1982). These conditions stimulate the growth of chemolithotrophic sulfide-oxidizing bacteria that oxidize the sulfide using oxygen or nitrate as terminal electron acceptor (Jørgensen, 1977; Robertson and Kuenen, 2006; Lavik et al., 2009). Gammaproteobacteria that oxidize sulfide in these settings include among others, the conspicuous vacuolated sulfur bacteria, *Beggiatoa* sp., *Thioploca*, and *Thiomargarita* sp. (Jørgensen and Gallardo, 1999; Schulz et al., 2000; Schulz and Schulz, 2005; Salman et al., 2011). Schulz and Schulz (2005) noted not just a regional correlation between the habitats of these sulfide-oxidizing bacteria, but also an intimate spatial association in the sediments between mats of *Thiomargarita namibiensis* and enrichment in pore water phosphate and apatite. Some microbes are known to accumulate phosphorus in high-energy polyphosphates that, when hydrolyzed as an energy reserve, may be expelled as phosphate, although polyphosphates also serve a wide variety of other cellular functions (Achbergerová and Nahálka, 2011). Schulz and Schulz (2005) demonstrated that *Thiomargarita* take up and store polyphosphate intracellularly. Through subsequent polyphosphate hydrolysis, they release enough phosphate as a pulse within a few centimeters of the sediment–water interface to account for the enriched pore water P and precipitation of apatite observed in *Thiomargarita*-inhabited sediments. Additional laboratory investigations of *Beggiatoa* by Brock and Schulz-Vogt (2011) suggest that exposure to sulfidic conditions initiates the utilization of stored polyphosphate in *Beggiatoa* and also perhaps in its close relative, *Thiomargarita*. Recently, Goldhammer et al. (2010) have documented the microbial uptake of ^33^P-labeled phosphate that rapidly passes from intracellular polyphosphate and into precipitated apatite, strengthening evidence of active microbial processing of P culminating in apatite precipitation. The isotope labeling experiments of Goldhammer et al. (2010) demonstrated phosphate sequestration in apatite occurring at a rate of 69–78 nmol cm^-^^2^day^-^^1^ under anoxic conditions, exceeding phosphate release from organic matter remineralization (9–36 nmol cm^-^^2^day^-^^1^). The rate of phosphate released by *Thiomargarita* in the laboratory was calculated to be sufficient to explain the mineral and pore water phosphate enrichment observed in phosphogenic sediments off Namibia (Schulz and Schulz, 2005). Thus, polyphosphate accumulating sulfide-oxidizing bacteria, experiencing alternating aerobic – anaerobic regimes, appear to be influential in focusing pore water phosphate where apatite precursors are actively forming. 

Other microbial processes might also be important for phosphate.enrichment in certain shallow marine sediments. Work by Arning et al. (2008),^ 2009^ attributes the build-up of pore water P to release from organics by sulfate-reducing bacteria, perhaps funneling P to polyphosphate-utilizing vacuolated sulfur bacteria. Investigations by Lucas and Prévot (1985) and Hirschler et al. (1990) present evidence of P accumulation by enzymatic breakdown of P-rich molecules like DNA, by alkaline phosphatase. It should be mentioned that polyphosphate utilization is by no means unique to sulfide-oxidizing bacteria, and considerable attention is given to the role of polyphosphate-accumulating bacteria in wastewater (Mino et al., 1987; Ohtake et al., 1998; Crocetti et al., 2000). Some bacteria not know to accumulate substantial polyphosphate, such as the common heterotrophic alphaproteobacterium, *Caulobacter crescentus* have, when cultured in the presence of high calcium concentration, precipitated minor amounts of carbonate hydroxyapatite (Benzerara et al., 2004), hence the processes that result in apatite precipitation in phosphogenic settings may be more complex than simple uptake and pulsed release of phosphate. Indeed, concentrations of phosphate in eutrophic lacustrine waters can become elevated (Penn et al., 2000), and yet the documented formation of calcium phosphate minerals in lake sediments is uncommon (Swirydczuk et al., 1981). Factors such as pH, redox potential (Eh), and the presence of other ions, as well as the existence of a suitable nucleation site and sufficient energy to activate precipitation, all have an effect on the rate and degree of precipitation. Microbial activity may influence these factors, particularly in fluids associated with microbial mats or heavily colonized sediments. For example, sulfide oxidation using oxygen as a terminal electron acceptor can produce acid:

$$H_{2}S\,\;+\;2O_{2}\,\to 2H^{+}+\;SO_{4}^{2-}$$

While sulfide oxidation with nitrate as the terminal electron acceptor consumes protons, whether it proceeds via denitrification:

$$5HS^{-}\,\;+\;8NO_{3}^{-}\,\;+3H^{+}\to 5SO_{4}^{2-}+4N_{2}+4H_{2}O$$

or dissimilatory nitrate-reducing ammonification:

$$HS^{-}\,\;+\;NO_{3}^{-}\,\;+H^{+}+H_{2}O\to SO_{4}^{2-}+NH_{4}^{+}$$

In addition to the effects of active microbial metabolisms, the cell ultrastructure can influence mineral precipitation. For example, charged cell walls and/or extracellular polymeric substances may bind ions, provide nucleation sites, and lower the activation energy required for precipitation (Ferris, 1989).

While considerable evidence supports the conclusion that polyphosphate utilization by sulfide-oxidizing bacteria contributes to apatite formation, this may not be the only microbial process involved in phosphogenesis. Though less commonly, phosphorite formation is known to occur in non-upwelling regimes (e.g., Ruttenberg and Berner, 1993) where the mechanisms for its deposition may differ markedly from those just described. For example, under anoxic conditions, dissimilatory iron reduction can dissolve iron-bearing minerals, releasing and concentrating adsorbed P in the pore water. Soluble reduced iron diffuses into bottom waters, precipitates, adsorbs more P, and sinks again, establishing an “iron-redox pump” whereby pore water P may be concentrated without direct concentration by microorganisms.

## MICROBES AND ANCIENT PHOSPHORITES

While microbes are thought to play a role in the apatite precipitation that leads to modern phosphorite formation, their role in ancient phosphogenesis is less clear. However, for many geological phenomena the present is the key to the past, and a number of microfossils and geochemical indicators suggest that microorganisms played an important role in the formation of ancient phosphatic deposits.

Phosphorites are known throughout much of the rock record, beginning with relatively minor phosphorites in the Paleoproterozoic (Papineau, 2010). Widespread volumetrically substantial phosphorites first occur during the Neoproterozoic-Cambrian transition, and afterward the occurrence of phosphorites is episodic and their abundances fluctuate substantially, with the Permian, Eocene, Miocene, and the Recent as intervals marked by substantial phosphorite formation (Cook, 1976; Cook and McElhinny, 1979; Cook and Shergold, 1986; Notholt and Sheldon, 1986; Riggs and Sheldon, 1990; Cook et al., 1990). Perhaps, the most prominent phosphogenic episode in Earth’s history occurred during the Neoproterozoic and continued across the Cambrian-Precambrian boundary ~600–550 Ma (Cook and Shergold, 1984). These phosphorites are associated with times of low-latitude glaciation, the spread of oxygen into benthic settings, the rifting of continents, sea level rise, and excursions of stable isotopes of sulfur and carbon (McFadden et al., 2008; Li et al., 2010; Planavsky et al., 2010). Many late Proterozoic and Cambrian phosphorites include episodes of black organic shales and pyritized materials, indicating at least locally sulfidic or stratified sulfidic conditions which may have supported extensive communities of sulfide-oxidizing bacteria (Cook and Shergold, 1984).

The proliferation of phosphorites across the Precambrian-Cambrian transition also occurs during an interval that records the origin and evolution of early metazoan life (Canfield et al., 2007). The ~600 Ma Doushantuo phosphorites of South China contain microfossils currently under study and variously interpreted as metazoan diapause-stage embryos, sulfide-oxidizing bacteria, and protists (Xiao et al., 1998; Bailey et al., 2007; Yin et al., 2007; Cunningham et al., 2011; Huldtgren et al., 2011). Following the Neoproterozoic-Cambrian phosphorite proliferation, phosphorite occurrences are episodic. Extensive phosphorites are known from the Permian, such as the Phosphoria Formation of the Western United States (Emigh, 1958; Cook, 1970); the Cretaceous to Eocene including the South Tethyan Phosphogenic Province (Pufahl et al., 2003); and the Miocene, including the Monterey Formation (Garrison et al., 1990) among others. The episodic timing of phosphorite deposition throughout geologic time may represent an interplay between the evolution of the geochemistry of marine waters, establishment of gradients between sulfidic and oxygenated waters, and the evolution of microbes able to exploit those geochemical conditions and gradients. Alternatively, or additionally, geologic intervals rich in phosphatic mineral deposits may simply represent relatively rare confluences of biological, sedimentological, tectonic, and ocean geochemical conditions resulting in massive phosphorite deposits (Filippelli, 2011).

**FIGURE 2:**
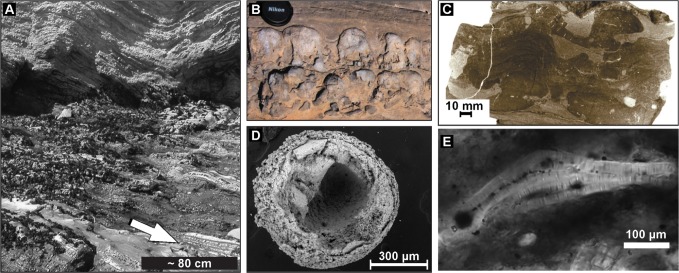
**(A)** Phosphorites (arrow) overlain by diatomites from the Miocene Monterey Formation at Shell Beach, California, preserve filaments in **(E)**. **(B)** Phosphatic microbialite in condensed horizon developed in OC-rich sandy mudstone horizon. Winnowed phosphate nodules are incorporated in the microbialite. **(C)** Intergrowth of columnar and flat-laminated phosphatic structures developed as a result of microbial mat growth and sediment phosphatization in OC-rich sandy mudstone. Vertical section, polished surface after staining to obtain color contrast between phosphatic and non-phosphatic sediments. **(D)** SEM of ~600 Ma Doushantuo microfossil showing layering reminiscent of cell membrane. **(E)** Bundle of large phosphatized filaments resembles filamentous sulfide-oxidizing bacteria. Credits: **(A)** and **(E)** from Bailey et al. (2009). **(B)** and **(C)** from Krajewski (2011).

 But what evidence is there that microbial activity influenced the deposition of ancient phosphorites? Possible apatite-bound bacterial microfossils in phosphorites were first described by Cayeux in 1936 and have since been found in phosphorite outcrops of many geologic ages (**Figure 2**) (Cayeux, 1936; Riggs, 1979; Soudry and Champetier, 1983; Williams and Reimers, 1983; Garrison and Kastner, 1990; Baturin et al., 2000; Soudry, 2000). Several workers have suggested that these possible bacterial fossils represent microbes capable of mediating phosphorite formation (O’Brian and Veeh, 1980; Williams and Reimers, 1983; Lamboy, 1990; Bailey et al., 2007). For example, the apparent remains of microbial mats within phosphate-rich laminations deposited below the photic zone in the Miocene Monterey Formation lead investigators to suggest that sulfide-oxidizing bacteria had been involved in phosphogenesis (Williams and Reimers, 1983; Williams, 1984; Reimers et al., 1990). However, the rather non-descript bacterial filaments put forward by Williams and Reimers (1983) include few morphological or geochemical features that could support their interpretation as the remains of sulfide-oxidizing bacteria. In addition to microfossils, phosphatic stromatolites provide possible evidence of the microbial mediation of phosphorite mineral precipitation (Banerjee, 1971; Krajewski et al., 2000). However, at present none of these microfossils or sedimentary structures contain strong diagnostic indicators that the phosphatized cells represent sulfide-oxidizing bacteria, or that the organisms preserved were involved in the phosphatization process. The identification of sulfide-oxidizing bacteria associated with the ancient phosphorite record has the potential to uncover the history of bacterial interactions with the marine phosphorous cycle, but diagnostic features that can be preserved in the ancient rock record will be required in order to conclusively identify them.

## CONCLUDING REMARKS AND UNANSWERED QUESTIONS

It seems that we are in the midst of a revolution in our understanding of the origins of phosphatic mineral deposits. Knowledge gained in fields as varied as wastewater treatment, geochemistry, and paleontology have all strengthened the argument for the role of microbes in the precipitation of apatite and the formation of phosphorite rock. A compelling mechanism for the concentration of phosphate in pore waters where apatite is actively precipitating has been discovered in the form of polyphosphate utilization by vacuolated sulfide-oxidizing bacteria. Many gaps remain in our understanding of the complete story. The processes and substrates involved in the transition from pore waters enriched in phosphate to the precipitation of apatite precursors remain poorly constrained. Eventual cultivation of vacuolated sulfide-oxidizing bacteria may allow for the experimental recreation of microbially mediated apatite precipitation in the laboratory. The rapid advancement of genomic and metagenomic approaches may bring us closer to understanding the genes involved in polyphosphate utilization, or help us understand community interactions in sediments where phosphogenesis is occurring.

Fossilized microbes have been identified in ancient phosphorites, and potentially some microfossils from ancient settings record the presence of sulfide-oxidizing bacteria. However, morphological criteria can frequently be insufficient for the identification of specific bacterial ecotypes. Future discovery and confirmation of evidence for microbially mediated phosphogenesis in the ancient rock record would be aided by the identification of diagnostic chemical signatures of microbes, such as lipid biomarkers, or signatures of the microbially mediated phosphatization process itself – perhaps a distinctive isotopic signature in phosphate-associated geochemical species. Additional investigation into the role of changing geological and oceanographic conditions in the production and preservation of ancient phosphorites, as well as sedimentary basin analysis, may reveal potentially relevant paleophysiographic features such as cratonic placement and orientation, or detailed isotopic characterization of phosphatic lithologies.

Phosphorite formation requires interactions among many aspects of the geosphere and biosphere, and a comprehensive understanding of the processes involved will require a multi-disciplinary approach applied to both modern phosphogenic environments and their ancient analogs. The results could have important ramifications for the exploration and sustainable management of this mineral resource so intimately connected to the global phosphorous cycle and to modern agriculture.

## Conflict of Interest Statement

The authors declare that the research was conducted in the absence of any commercial or financial relationships that could be construed as a potential conflict of interest.
